# Nanotechnology-based drug delivery of ropinirole for Parkinson’s disease

**DOI:** 10.1080/10717544.2017.1359862

**Published:** 2017-08-06

**Authors:** Emilia Barcia, Liudmila Boeva, Luis García-García, Karla Slowing, Ana Fernández-Carballido, Yaquelyn Casanova, Sofía Negro

**Affiliations:** a Departamento de Farmacia y Tecnología Farmacéutica, Facultad de Farmacia, Universidad Complutense de Madrid, Madrid, Spain;; b Unidad de Cartografía Cerebral, Instituto Pluridisciplinar, Universidad Complutense de Madrid, Madrid, Spain;; c Departamento de Farmacología, Facultad de Farmacia, Universidad Complutense de Madrid, Madrid, Spain

**Keywords:** Ropinirole, nanoparticles, poly (D,L-lactide-co-glycolide), Parkinson’s disease

## Abstract

A new drug delivery system is developed for ropinirole (RP) for the treatment of Parkinson’s disease (PD) consisting of biodegradable poly (D,L-lactide-co-glycolide) (PLGA) nanoparticles (NPs). The formulation selected was prepared with 8 mg RP and 50 mg PLGA 502. This formulation exhibited mean encapsulation efficiency of 74.8 ± 8.2%, mean particle size lower than 155 nm, the zeta potential of −14.25 ± 0.43 mV and zero-order *in vitro* release of RP (14.13 ± 0.17 μg/h/10 mg NPs) for 5 d. Daily doses of the neurotoxin rotenone (2 mg/kg) given i.p. to male Wistar rats induced neuronal and behavioral changes similar to those of PD. Once neurodegeneration was established (15 d) animals received RP in saline (1 mg/kg/d for 35 d) or encapsulated within PLGA NPs (amount of NPs equivalent to 1 mg/kg/d RP every 3 d for 35 d). Brain histology and immunochemistry (Nissl-staining, glial fibrillary acidic protein and tyrosine hydroxylase immunohistochemistry) and behavioral testing (catalepsy, akinesia, rotarod and swim test) showed that RP-loaded PLGA NPs were able to revert PD-like symptoms of neurodegeneration in the animal model assayed.

## Introduction

Parkinson’s disease (PD) is after Alzheimer’s the second neurodegenerative disorder in prevalence (Tolosa et al., 2006). PD is a progressive disorder of the nervous system which affects movement being characterized by motor alterations such as bradykinesia, rigidity, postural instability as well as non-motor symptoms due to significant loss of dopaminergic neurons.

Ropinirole (RP) is a non-ergolinic dopaminergic agonist exhibiting high affinity for both D2 and D3 receptors (Jost & Angersbach, 2005; Jost et al., 2008). It is indicated as monotherapy in early PD and as adjunctive therapy to levodopa. RP is given orally as immediate-release (IR) tablets taken three times daily or as extended-release (ER) tablets taken once daily. When RP formulated in IR tablets is given orally the drug is rapidly and almost completely absorbed. However, due to extensive first-pass hepatic metabolism oral bioavailability only ranges from 36% to 57% and plasma elimination half-life is short (5–6 h) (Bhatt et al., 2011). Its low bioavailability limits the amount of RP that could gain access to the CNS. The usage of sustained drug delivery systems have some advantages such as the reduction of dosing frequency which in turns can result in lower adverse effects, enhanced therapeutic efficacy and with the possibility of preventing the first-pass metabolism. However, in the case of RP, when ER tablets are used, the systemic exposure obtained is comparable to that of IR formulations (same daily doses), which makes relative bioavailability of RP from ER tablets similar to that of IR tablets (Tompson & Vearer, 2007; Stocchi et al., 2008). In both cases (IR and ER tablets) most of RP does not reach the brain due to first-pass hepatic metabolism. A study conducted by Tompson & Vearer (2007) indicated that administration of RP as once-daily ER tablets may produce nocturnal concentrations lower than those obtained with the three times daily IR formulation. The nocturnal ‘off’ symptoms could also be more severe with the ER tablets when compared with the IR formulation. Moreover and according to Yun et al. (2013), the early morning ‘off’ period could also be longer when using ER tablets since a smoother increase of RP plasma levels was obtained with this formulation.

When treating PD novel therapeutic strategies that focus on sustained drug release systems are being investigated to achieve continuous dopaminergic stimulation. Moreover, the development of new drug delivery systems able to cross the blood brain barrier (BBB) can be very interesting when searching for new therapeutic strategies for neurodegenerative diseases such as PD (Kasinathan et al., 2015).

The BBB is a highly selective permeable barrier which represents the main obstacle for drugs to enter the CNS. This barrier is composed of non-fenestrated endothelial cells, linked by tight junctions, two basement membranes and astrocytic end-feet. The tight junctions between the endothelial membranes almost completely seal the BBB thereby limiting the penetration of circulating endogenous and exogenous compounds (Serlin et al., 2015). For this, much effort is being currently conducted to improve drug transport across the BBB and into the CNS.

Among the strategies used to gain access to the brain polymeric nanoparticles (NPs) are being investigated due to the fact that they provide protection for therapeutic agents while efficiently delivering them into damaged areas of the brain (Linazasoro, 2008; Spuch et al., 2012; Marcianes et al., 2017).

The present study is aimed to the development and characterization of biodegradable RP-loaded NPs to target the drug to its site of action. The formulations developed are characterized and evaluated in a rotenone (RT)-induced animal model of PD. RT is a natural neurotoxicant used as a pesticide (Alam & Schmidt, 2002). When administrated to experimental animals RT is able to induce some of the behavioral, neurochemical and neuropathological changes occurring in PD (Sherer et al., 2003). To this date, the mechanism involved is not fully understood, although it has been reported that RT is capable of inhibiting the mitochondrial complex I (NADH-ubiquinone reductase), showing a relative specificity for dopaminergic neurons. This mechanism is shared with other neurotoxicants known to induce parkinsonism such as 1-methyl-4-phenyl-1,2,3,6-tetrahydropyridine (MPTP) (Schapira, 1994). Such mitochondrial impairment is present in human patients suffering from the non-familial forms of PD (Schapira et al., 1989). This finding supports the proposition that PD may be due to environmental toxins with actions similar to those of MPTP and RT. Other actions have been reported to participate in the neuronal damage induced by RT, including oxidative stress, microglial activation, production of reactive oxygen species (ROS), apoptosis and α-synuclein aggregation (Uversky, 2004).

## Materials and methods

### Materials

RP-free base was obtained from Zhejiang Medicine & Health Products Import & Export Co., Ltd. (China); RT was purchased from Sigma-Aldrich (Madrid, Spain); Poly (D,L-lactide-co-glycolide) (PLGA) Resomer^®^ RG 502, 50:50, and inherent viscosity 0.2 dl/g was purchased from Evonik Industries (Darmstadt, Germany). Polyvinyl alcohol (PVA) (*M*_w_ = 72 kDa) was obtained from Merck (Madrid, Spain). All other reagents and solvents were of analytical grade and provided by Panreac (Barcelona, Spain). Water was purified in a Milli-Q filtration system (Millipore, Billerica, MA) and used in the preparation of buffers and solutions.

### Preparation of RP-loaded PLGA nanoparticles

RP-loaded PLGA NPs were prepared by nanoprecipitation. The nanoprecipitation method was selected since it required lower external energy and allowed us to use acetone which exhibits lower toxicity than other solvents (Katiyar et al., 2016).

For method optimization, the following amounts of RP were assayed: 4.5; 5; 8 and 10 mg. In all cases, 50 mg of PLGA 502 were used in the preparation of NPs. Briefly, RP and PLGA 502 are dissolved in 4 ml acetone under vortex agitation for 2 min. The dissolution formed is added to 12 ml of 0.5% PVA under continuous stirring for 15 min to obtain the NPs. Then, the suspension formed is evaporated in a rotavapor for 2 h at 25 °C and 70 mbar (Buchi Rotavapor-R, BÜCHI Labortechnik AG, Flawil, Switzerland) to completely remove acetone. The resulting suspension is washed three times with distilled water and centrifuged (Avanti J-301, Beckman Coulter Inc., Brea, CA) at 15,000 rpm for 30 min to remove all PVA. Then the dispersed solution obtained is freeze-dried for 24 h using 3% sucrose as a cryoprotectant (Flexi-Dry MP™, FTS^®^ Systems, Stone Ridge, NY).

### Characterization of nanoparticles

#### Morphological characterization and size distribution

The morphology of the NPs was analyzed by emission scanning electron microscopy (JEOL JEM 6335F, Jeol Ltd., Tokyo, Japan). Samples were coated with a thin layer of colloidal gold applied in a cathodic vacuum evaporator before observation by SEM at 20 kV. The mean diameter and size distribution of the particles were evaluated by laser diffraction using a Microtrac-S3500 particle size analyzer (Microtrac S3500, Microtac Inc., Montgomeryville, PA) at 25 °C. The lyophilized samples were suspended in Milli-Q water and sonicated for 30 s before each determination to prevent clumping. Results are described in terms of mean diameter as well as standard deviation.

#### X-ray diffraction (XRPD)

X-ray diffraction patterns were measured in an automatic powder Philips X-Pert MPD diffractometer (Panalytical, GH Eindhoven, Netherlands) combined with a high-temperature chamber (Anton Paar HTK 10) with a Pt heating filament, Ni-filtered Cu–K_α_ irradiation (*λ* = 1.542 Å), a 2*θ* interval configuration, angle range 5–40°, scan step size 0.04° and time per step 1 s. Under these conditions, samples of RP, polymer, blank NPs and RP-loaded NPs were assayed.

#### Infrared absorption spectrophotometry (IR)

IR spectra were recorded on Fourier transform infrared (FTIR) spectra with a Thermo Nicolet Nicolet Nexus spectrophotometer (Thermo Scientific, Waltham, MA) equipped with the Goldengate accessory for attenuated total reflection (ATR). Scans were recorded at a resolution of 2 cm^−1^ over the wave number region 400–4000 cm^−1^. IR spectra were obtained for RP, PLGA, blank NPs and RP-loaded NPs.

#### Determination of process yield and encapsulation efficiency

Determination of process yield (%) was made from the ratio between the total weight of NPs obtained and the total weight of both drug and polymer used. Calculation of encapsulation efficiency (EE%) was done from the ratio between the amount of RP content in the NPs and the amount of drug used for their preparation. For this, 10 mg of NPs were dissolved in 1 ml CH_2_Cl_2_. Then the polymer was precipitated by adding ethanol (5 ml). The supernatant obtained after centrifugation (Universal 32, Hettich, Germany) at 5000 g for 5 min was filtered through 0.45 mm filters and analyzed by the HPLC method developed and validated by the authors (Fuster et al., 2015). The apparatus consisted in an HPLC A Series 200 PerkinElmer chromatograph equipped with a 1740 HP computer and a 235 C diode array detector (PerkinElmer, Waltham, MA). A Kromasil 100 column (250 × 4 mm, 5 µm) (Teknokroma, S. Coop., Barcelona, Spain) was used with a mobile phase consisting of acetonitrile:phosphate buffer (55:45, v/v), adjusted to pH 6 and containing 0.3% triethanolamine. Flow rate was set at 1 ml/min, the injection volume was 20 μl and the detection wavelength 245 nm. All analyses were performed at 25 ± 0.5 °C with each determination made in triplicate. The HPLC method was validated to demonstrate the absence of interference between RP and PLGA.

#### Zeta potential

Zeta potential was evaluated in a laser-Doppler anemometry using a Malvern Zetasizer (Malvern Instruments, Worcestershire, UK) with all determinations performed at 25 °C in aqueous media. The effective voltage used was 150 V. For zeta potential determinations, 5 mg of each formulation was weighed, placed in a flask, diluted with 50 ml distilled water and maintained in a sonicator for 5 min. Then the samples were introduced into a capillary cell (Cell Enhances Capillary^®^, Malvern Instruments), for zeta potential measurements. All formulations were analyzed in triplicate.

#### *In vitro* drug release study

*In vitro* release of RP from the NPs was investigated. For this, NPs (10 mg) was suspended in 3 ml of ethanol/water (40:60) (sink conditions) in a water bath (Memmert, Germany) at 37 ± 0.2 °C and constant agitation (100 rpm). At predetermined time intervals all volume was withdrawn, the supernatant extracted, filtered through 0.45 μm filters and replaced with the same volume of fresh medium. *In vitro* release tests were carried out for 6 d. Quantification of RP was performed by the HPLC method indicated before. *In vitro* release tests were done in triplicate.

### Animal testing

Male Wistar rats (Harlan, France), weighing 180–220 g at the beginning of the experiment were used in this study. Animals were housed at 22 ± 2 °C under normal laboratory conditions and on a standard light–dark cycle. Food and water were supplied *ad libitum*. All experiments were carried out according to the European Communities Council Directive of November 24 1986 (86/609/EEC) regarding the use and care of animals for experimental procedures. To minimize pain and discomfort all adequate measures were taken with efforts made to minimize the number of animals used.

#### Treatments and animal groups

**Scheme 1.**
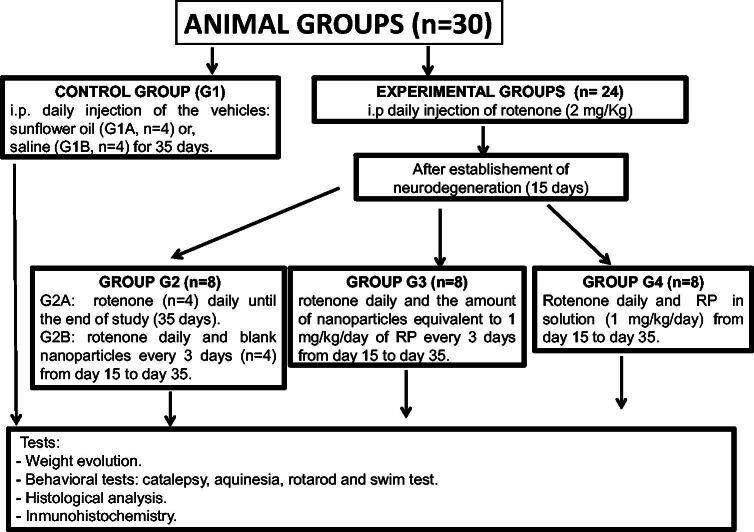
Experimental animal groups.

The neurotoxin used (RT) was dissolved in sunflower oil before administration (Fernández et al., 2012). Animals were divided into the following five groups each containing eight animals (Scheme 1):

- Group 1 (G1): Control group. Animals (*n* = 8) receiving the vehicles; sunflower oil (group G1A, *n* = 4) or saline (group G1B, *n* = 4).
- Group 2 (G2): Animals (*n* = 8) receiving RT (2 mg/kg/d) for 35 d (G2A) (*n* = 4) or RT (2 mg/kg/d) and blank NPs every 3 d from day 15 (G2B, *n* = 4).
- Group 3 (G3): Animals (*n* = 8) receiving RT (2 mg/kg/d) for 35 d and the amount of NPs equivalent to 1 mg/kg/d RP every 3 d from day 15.
- Group 4 (G4): Animals (*n* = 8) receiving RT (2 mg/kg/d) for 35 d and RP in saline (1 mg/kg/d) from day 15.

The selection of the doses assayed was done based upon previous experiments carried out in our laboratory. For this, appropriate amounts of NPs adapted to animal weight and EE were injected intraperitoneally. RT was given in sunflower oil and the NPs in saline. After 36 d animals were sacrificed by decapitation with a guillotine.

#### Body weight evaluation

On pre-established days (day 1, 5, 10, 15, 20, 25, 30 and 35) animals were weighed to evaluate changes occurring throughout the study.

#### Behavioral testing

##### Catalepsy test

Catalepsy test (grid and bar) was carried out at pre-determined times (days 15, 22, 29 and 36). For the grid-part of the test animals were hung by all four paws on a vertical grid (25.5 × 44 cm, with a space of 1 cm between each wire), and as soon as the animal showed the first movement the time was recorded as descent latency. For the bar-part of the test, animals were placed with both forepaws on a bar which was 10 cm above and parallel from the base. The rats were placed with both forepaws on the bar in a half-rearing position. Latency with the removal of the paw was noted. The maximum descent latency was fixed at 180 s for both tests. Catalepsy tests were carried out in triplicate for each animal.

##### Akinesia test

Akinesia test was performed at pre-determined times (days 15, 22, 29 and 36) and determining the latency (seconds) of the animals employed to move all four limbs. The test finished when latency exceeded 180 s. Animal adaptation to the test was achieved by placing them for 5 min on an elevated (100 cm) platform (100 × 150 cm). Afterward, the time taken by the animal to move all the four limbs was recorded. Akinesia tests were performed in triplicate for each animal.

##### Rotarod

A rotarod system (Rotarod LE 8200; Letica Scientific Instruments, Barcelona, Spain) equipped with automatic timers and falling sensors was used to evaluate motor balance and coordination in the animals. Adaptation to the rotarod was previously performed on five consecutive days by placing the animals for 1 min on the system. For the test, animals were placed on the rotarod at a constant speed (12 rpm) and the time (latency in seconds) taken for the animals to fall from the rotarod was recorded. The test finished when latency exceeded 300 s. Rotarod tests were carried out in triplicate on days 15, 22, 29 and 36.

##### Swim-test

Swim-tests were carried out on days 15, 22, 29 and 36 of the study, according to the procedure described by Haobam et al. (2005) which was adapted to our experimental conditions. The test was performed in water tubs (40 × 40 × 40 cm) with the temperature maintained at 27 ± 2 °C. Swim-scores are as follows: 0, hind part sinks with head floating; 1, occasional swimming using hind limbs while floating on one side; 2, occasional floating/swimming only; and 3, continuous swimming. Tests were performed in triplicate for each animal.

#### Histochemical assessments

##### Brain processing

At the end of the study (36 d) animals were sacrificed by decapitation. Immediately after decapitation brains were removed, frozen on dry ice and stored at −80 °C until analysis. Coronal brain sections (30 μm thick) at the level of striatum and substantia nigra were obtained by means of a cryostat (Leica CM1850, Leica Biosystems, Wetzlar, Germany). All brain slices were thaw-mounted onto Superfrost Plus slides (Thermo Scientific, Karlsruhe, Germany), dried at 36 °C on a hot plate and kept frozen at −80 °C.

##### Nissl-staining

For the Nissl-staining brain, slices were fixed in 4% formaldehyde in phosphate buffer at pH 7.4. Samples were then washed twice with phosphate buffer, and submerged in 0.5% cresyl violet acid solution for 30 min. Afterwards, samples were washed in distilled water and dehydrated in graded ethanol solutions (70%, 95% and 100%). The slices were finally cleared in xylene (twice, 5 min each). Then, the slices were coverslipped with dibutylphthalate polystyrene xylene (DPX) organic mounting medium (Sigma-Aldrich, Spain). Once dried, histological images at the level of substantia nigra were obtained with a digital camera (DFC425 camera, Leica) coupled to a light microscope (Leitz Laborlux S, Leica).

##### Glial fibrillary acidic protein and TH immunohistochemistry

Glial fibrillary acidic protein immunohistochemistry was carried out as previously reported by García-García et al. (2015) with minor modifications. The samples were fixed with 4% formaldehyde, washed, permeabilized with tris-buffered saline (TBS)/0.1% Tween 20 and blocked with 5% albumin dissolved in TBS. Then, the slides were incubated overnight at 4 °C with a fluorescent anti-GFAP antibody conjugated with the cyanine dye Cy3 (dilution 1:500; Sigma-Aldrich, St. Louis, MO), eliminating the need to use a secondary antibody. After washing for removal of the unbound antibody, the brain sections were cover-slipped with the homemade Mowiol aqueous mounting medium. Digital images were then obtained with a fluorescence digital camera (Leica DFC 3000G) coupled to a microscope (Leica DM2000LED) using tetramethylrhodamine isothiocyanate filter.

For tyrosine hydroxylase (TH) immunohistochemistry, a standard procedure was followed. After fixing, permeabilizing with 0.1% Tween 20 in TBS, washing and blocking, the TH antibody (dilution 1:500, Santa Cruz Biotechnology, Heiderberg, Germany) was added to the samples. After overnight incubation at 4 °C, the slides were washed with TBS/0.1% Tween 20 (3 × 5 min at RT) and the corresponding secondary FITC-labeled antibody (dilution 1:500; Santa Cruz Biotechnology) was then added. Finally, after incubation (2 h at RT) and washing, the slides were mounted with Mowiol aqueous medium and observed with a fluoresce microscope (Leica DM2000LED). Digital images were captured using the FITC filter (Leica DFC 3000G).

### Statistical analysis

Data are expressed as mean ± standard error of the mean (SEM). Statistical analysis was performed by one-way ANOVA. Statistical significance was defined as *p* < .05. Statistical analyses were carried out using the Statgraphics^®^ *Plus* version 5.1 software (John Wiley and Sons, Oxford, UK). The catalepsy, akinesia, rotarod and swim results were analyzed for statistical significance employing non-parametric analyses (multifactorial Kruskal–Wallis one-way ANOVA).

## Results and discussion

Nowadays, there still exists the need for new treatments or an improvement of the currently available treatments for PD. NPs are considered one of the most promising and versatile drug delivery systems to improve the access of drugs into the CNS and to reduce side-effects. For this, the present study was aimed to develop a new delivery system for RP consisting of PLGA NPs.

Different RP-loaded PLGA formulations have been prepared with different amounts of RP (Table 1, formulations NPRP-1 to NPRP-4). Mean diameters of NPs as measured by dynamic light scattering were lower than <200 nm (Table 1), being therefore suitable for the purpose of crossing the BBB. Several studies have demonstrated that polymeric NPs made with biodegradable polymers such as PLGA and with particle sizes around 250 nm, are able to reach different areas of the brain (Hillaireau & Couvreu, 2009; Wohlfart et al., 2012). For all formulations, the polydispersity index was lower than 0.5 indicating a slight polydispersion in particle sizes (Table 1) (Marcianes et al., 2017).

**Table 1.** Formulations prepared.

Formulation	Amount of RP (mg)	Particle size (nm) (mean ± SD)	Polydispersity index (mean)	Yield of production (%) (mean ± SD)	Encapsulation efficiency (%) (mean ± SD)
NPRP-1	4.5	148.4 ± 4.1	0.31	47.3 ± 2.7	54.5 ± 7.7
NPRP-2	5	133.9 ± 5.7	0.45	50.1 ± 3.5	65.5 ± 0.9
NPRP-3	8	152.2 ± 3.1	0.29	56.7 ± 5.5	74.8 ± 8.2
NPRP-4	10	155.0 ± 4.0	0.28	48.9 ± 7.9	58.14 ± 10.4

RP (ropinirole).

The mean values of process yield for all RP-loaded PLGA NPs ranged from 47.3 ± 2.7% to 56.7 ± 5.5% (Table 1). EE increased as the amount of RP increased from 4 to 8 mg; however, this increase was not observed when 10 mg RP was used. The highest value of EE was obtained for the formulation made with 8 mg of RP (74.8 ± 8.2%). The drug loading of RP into the NPs was due to the partitioning of the drug dispersed in the organic phase into the external phase. High drug payloads are advantageous to minimize the amount of NPs administered and to reduce the amount of polymer given (Reis et al., 2006a,b). Taking into consideration all the results obtained formulation NPRP-3 was selected to continue the study.

**Figure 1.**
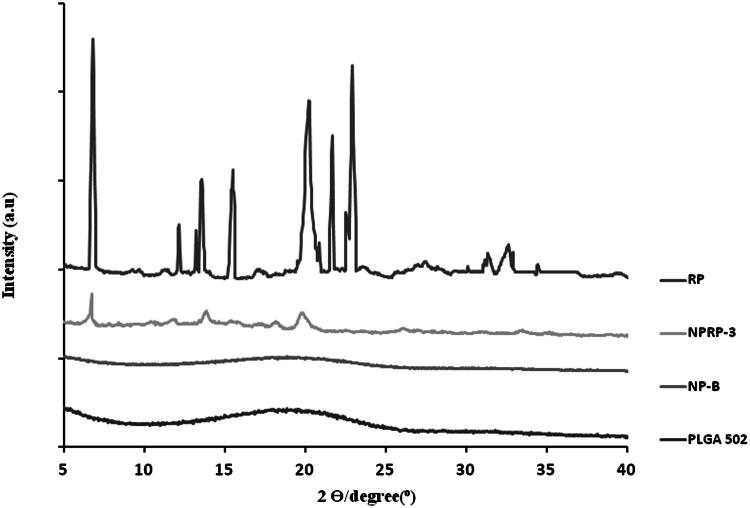
X-ray scattered intensity as a function of the diffraction angle 2θ. Intensity scaling is arbitrarily applied to the curves. Curves obtained for polymer (PLGA 502), blank nanoparticles (NP-B), RP-loaded nanoparticles (NPRP-3) and ropinirole (RP).

The X-ray Powder Diffractometry (XPDR) diffraction pattern of RP was characteristic of a crystalline state with intensity maxima appearing at 6.82°, 20.02°, 21.70 and 22.86° (Figure 1). The diffraction pattern obtained for PLGA 502 showed no maximum which confirms its amorphous state indicating that the encapsulation technique used had no effect on the polymer characteristics. The pattern obtained for formulation NPRP-3, prepared with 8 mg RP showed the characteristic peaks of RP thereby indicating that the drug is partially in a crystalline state.

**Figure 2.**
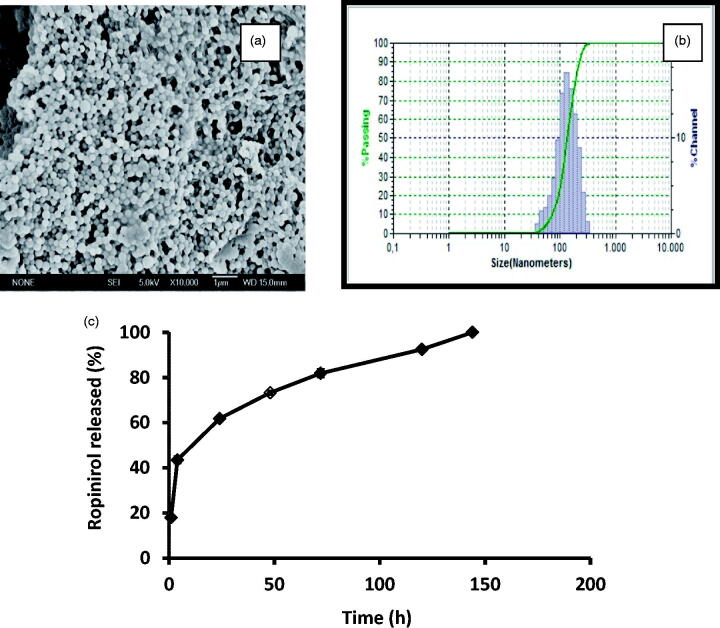
SEM microphotographs of RP-loaded PLGA nanoparticles (a). Particle size distribution (DLS image) (b). Mean release profiles (± S.E.M., n=3) of RP from PLGA nanoparticles (c). RP (ropinirole).

**Figure 3.**
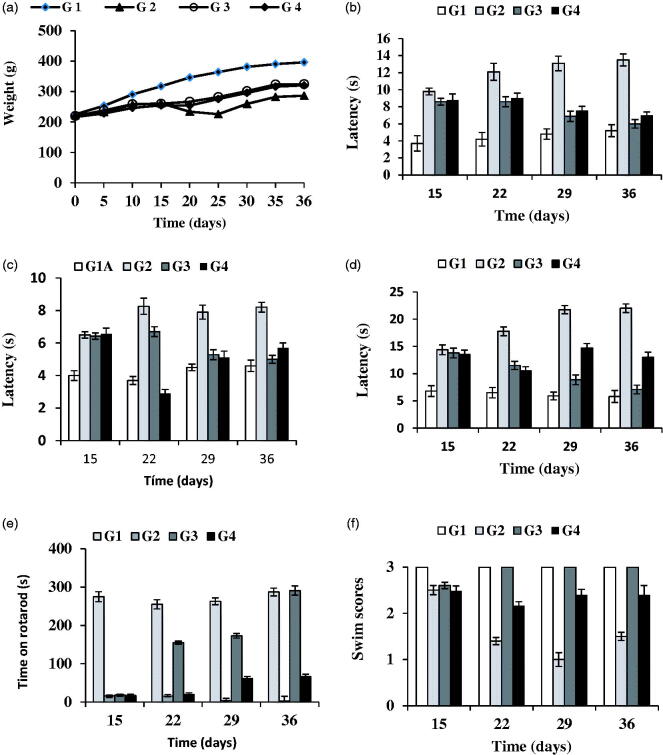
Evolution of rat-body weight throughout the study (36 d) (a). Results of the behavioral tests: catalepsy test on bar (b) and grid (c), akinesia test (d), rotarod (e) and swim-test (f) performed in all animal groups. G1: control group; G2: RT-treated control group; G3 and G4 RT-treated animals also receiving; G3: RP-loaded PLGA nanoparticles; and G4: RP in saline. RT (rotenone); RP (ropinirole).

FTIR spectrum of PLGA (not shown) is consistent with the expected structure of the copolymer showing a strong peak at 1720 cm^−1^ which is assigned to C = O stretch, absorption at 1270-1150 cm^−1^ due to asymmetric and symmetric C-C(=0)-0, the bands in these regions are characteristics of esters groups and also a peak appearing at 1045 cm^−1^ which correspond to C-O. The spectrum corresponding to blank NPs did not show any changes with respect to that of PLGA alone thereby indicating that the encapsulation procedure did not modify the polymer characteristics. Nevertheless, a broad band in the region of 3500–3200 cm^−1^ was observed, probably due to the OH groups of PVA which was used for the preparation of the NPs. When RP was analyzed a peak corresponding to NH appeared at 3420 cm^−1^, a strong band was observed at 3100-2750 cm^−1^ due to C-H as well as some peaks in its fingerprint region (1700-600 cm^−1^) which are somehow overlapped by those of PLGA. Analysis of RP-loaded NPs showed similar peaks to those obtained for PLGA, PVA and RP. However, due to the drug-polymer ratio used in the preparation of the selected NP formulation (NPRP-3, Table 1) the fingerprint region of RP is to a certain extent masked by the spectrum of PLGA.

Determination of the zeta potential of nanosystems is of great interest due to its influence on the stability of the suspension and on their passage through the BBB. Positively charged NPs may interact with negatively charged cell surfaces thereby representing an advantage, but a stronger immune response is obtained. On the other hand, negatively charged particles at low concentrations do not alter the BBB (Lockman et al., 2004). In our case, the value of zeta potential for the formulation selected (NPRP-3) was −14.25 ± 0.43 mV which is adequate for crossing the BBB.

Figure 2 shows microphotographs of formulation NPRP-3, an example of its particle size distribution (DLS image) as well as the *in vitro* release profiles obtained for RP from this formulation. As it can be seen a burst release of around 20% is obtained within the first 24 h followed by a slower release for 5 d that can be described by a mean zero-order release rate constant of 4.13 ± 0.17 μg/h/10 mg NPs.

**Figure 4.**
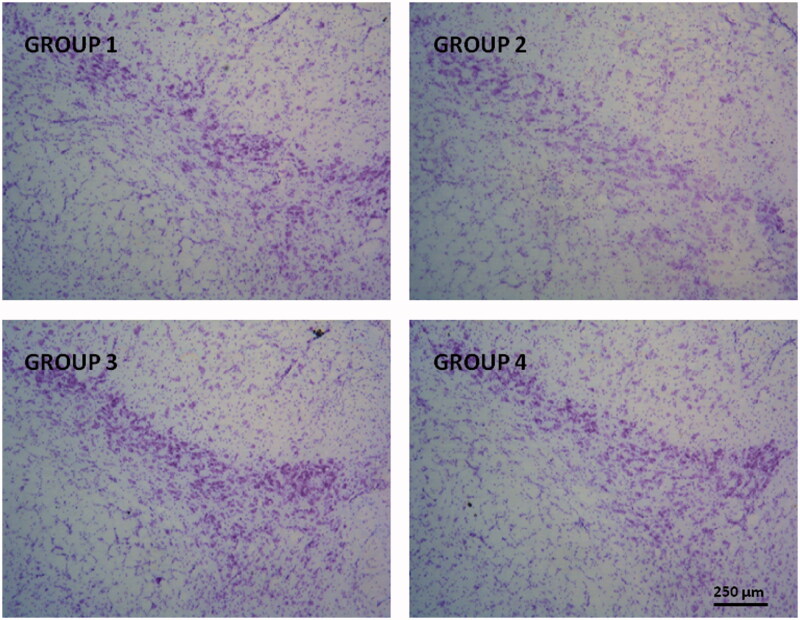
Representative Nissl-staining (cresyl violet) of nigral neurons from brain sections (substantia nigra pars compacta, 30** **µm) corresponding to all animal groups. G1: control group; G2: RT-treated control group; G3 and G4 RT-treated animals also receiving; G3: RP-loaded PLGA nanoparticles and G4: RP in saline; RT: rotenone; RP: ropinirole.

**Figure 5.**
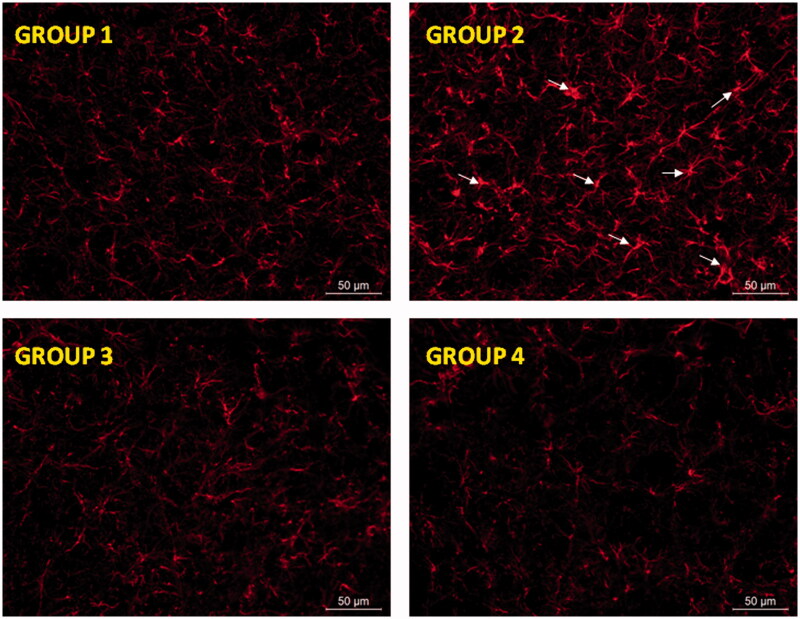
Glial activation induced by RT at the level of substantia nigra was prevented by RP as assessed by GFAP immunohistochemistry. Representative fluorescence micrographs of coronal brain slices at the level of the substantia nigra from all animal groups assayed (G1–G4). Arrow heads point to aberrant cell structures. G1: control group; G2: RT-treated control group; G3: RP-loaded PLGA nanoparticles and G4: RP in saline. RT: rotenone; RP: ropinirole.

To evaluate the efficacy of the new nanosystem developed the selected formulation was tested in an RT-induced animal model of PD. RT is able to induce various features of PD in animals including Lewy body formation in the nigral neurons causing a gradual and uniform damage of the nigrostriatal system (Sindhu et al., 2005).

To evaluate the therapeutic efficiency of the new formulation behavioral, histological and immunochemistry analyses were conducted. Behavioral testing assists in the study of symptom-relieving effects of therapy, as behavioral tests can be translated into clinical performance in human cases. In addition, histological and immunochemistry evaluation helps to study the improvement of diseases following treatment.

In our study, mortality occurred in 14.3% of the animals treated with RT with no deaths occurring in control animals. Other authors (Cannon et al., 2009) using RT dissolved in DMSO (2.75–3 mg/kg/d) reported 10% mortality occurring in animals shortly (minutes) after injection. Zhang et al. (2017) evaluated RT at two dose levels (2 and 2.5 mg/kg) obtaining 46.7% mortality at the highest dose when compared with only 6.7% at the lower dose assayed. In our case, none of the animals died shortly after injection probably due to the lower dose of RT employed (2 mg/kg/d) and the lower toxicity of the vehicle used in our study (sunflower oil).

The evolution of body weight was recorded at times 0, 5, 10, 15, 20, 25, 30 and 35 d (Figure 3(a)). Animals belonging to control groups (G1A and G1B), which correspond to those receiving the vehicles (sunflower oil or saline) experienced a gradual and steady weight gain throughout the study with non-significant differences (*p >* .05) observed between both control groups (G1A and G1B) and subgroups G2A and G2B. Animals treated with RT (groups G2 and G3) showed a very slight weight increase during the first 2 weeks, probably due to the development of neurodegeneration. After 15 d animals treated with RP (groups G3 and G4) constantly improved their weight gain in contrast with group G2.

The results obtained in the catalepsy test (gird and bar) at 15, 22, 29 and 36 d are shown in Figure 3(b,c). Non-significant differences (*p* > .05) were obtained in control animals treated with both vehicles; sunflower oil (G1A) or saline (G1B). However, statistically significant differences were found between RT-treated animals (G2) and control animals (G1A and G1B). The neurotoxin RT induced an increase in latency both in the grid and bar tests of catalepsy which was reverted by RP. Administration of RP in saline or encapsulated within NPs clearly improved latency from day 22 of the study which corresponds to 7 d after initiating the administration of RP. However, at the end of the study period (36 d), the best results were achieved in group G3 which received RP-loaded PLGA NPs, thereby confirming the potential interest of the new nanosystem developed for RP.

Figure 3(d) shows the results obtained for the akinesia test in which non-significant differences were found in both control subgroups (G1A, G1B). The administration of RT (group G2) significantly prolonged descent latency when compared with G1A and G1B, with a value of latency 2.5 times higher in group G2 with respect to those of G1A or G1B. As in the catalepsy test, the best results corresponded to group G3 receiving RP-loaded NPs. At the end of study (36 d), the latency values for group G3 were similar to those of control subgroups (G1A, G1B). However, administration of RP in saline (1 mg/kg/d) resulted at the end of the study in latency values which were statistically significant higher than those of control group (G1A, G1B).

The rotarod test is widely used to evaluate motor coordination in rodents being especially sensitive in detecting cerebellar dysfunction. The rotarod performance is affected by several factors such as lack of coordination, bradykinesia and rigidity. This test was performed on days 15, 22, 29 and 36. The administration of RT produced a significant reduction in the time that animals spent on the rotarod when compared with control group (G2 versus G1A or G1B) (Figure 3(e)). These marked differences are indicative of the neurodegeneration produced by the neurotoxin. It has been recently indicated that local injection of RT into the substantia nigra pars compacta (SNpc), as well as chronic subcutaneous injections of RT, resulted in disturbed behavior on the rotarod (von Wrangel et al., 2015). The administration of RP (groups G3 and G4) improved motor coordination with the best results obtained for group G3. These animals showed at the end of study similar results to control animals.

Figure 3(f) shows the results obtained in the swim-test which is used to monitor the overall motor ability/deficit of the animal. At all times assayed control animals (G1A and G1B) obtained the highest values (score = 3) (Figure 3(f)). Swimming ability of RT-treatment animals (group G2) was decreased when compared to control animals (*p* >.05) (Figure 3(f); G2 versus G1A or G1B). Animals receiving RP (groups G3 and G4) showed higher swimming scores than group G2. As with the other behavioral tests, the best results corresponded to group G3. For these animals swimming scores after 22 d were similar to those of control animals.

Dopaminergic agonists such as RP and pramipexole are able to reduce the symptomatology of PD as well as to protect against RT-induced neurotoxicity (Schapira, 2002; Chen et al., 2008). Several mechanisms seem to intervene in the neuroprotective effects exerted by RP in different experimental models of PD, including reduction of both apoptosis markers and neuroinflammation (Park et al., 2013), and partial restoration of the antioxidant balance (Bisht et al., 2014).

Nissl-staining is a histological method frequently employed to analyze the morphology and pathology of neurons as well as to study the cytoarchitectony of the brain (Pilati et al., 2008). The results obtained in our study showed signs of neuronal damage at the level of the SNpc in RT-treated animals with an observable reduction in the number neurons (Figure 4, group G2). These results are in agreement with those reported by other authors (Swarnkar et al., 2013; Tapias et al., 2013). Subchronic treatment with RP significantly prevented the RT-induced cell death at the level of SNpc, regardless the formulation used (Groups G3 or G4), although a slight higher neuroprotective effect was observable when RP was given as NPs.

**Figure 6.**
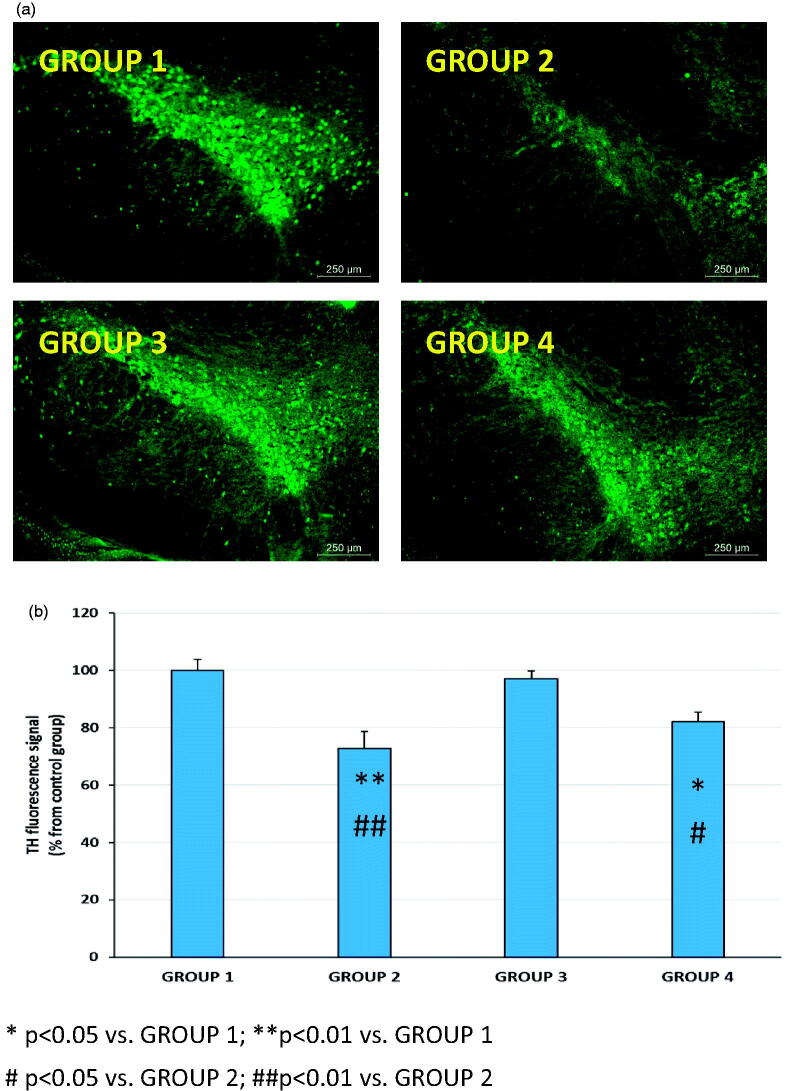
Representative TH immunohistochemistry fluorescence (a) and TH fluorescence signal (b) in coronal brain slices at the level of the substantia nigra for all animal groups (G1 to G4). G1: control group; G2: RT-treated control group; G3: RP-loaded PLGA nanoparticles; and G4: RP in saline. RT (rotenone); RP (ropinirole).

Neurodegeneration is usually associated with activation of glial cells playing an essential role for the development of neuronal damage by increasing the generation of ROS (Thomas et al., 2004). Moreover, when a brain insult occurs a slow astrocytic response can maintain microglia activation and eventually lead to a chronic brain lesion (Gao et al., 2013). When using RT as neurotoxin astrocytic activation has been recently reported both *in vitro* (Swarnkar et al., 2012) and *in vivo* (Tapias et al., 2013). As Figure 5 shows, an intense astroglial response in the substantia nigra was observed after RT treatment. This astrocytic response was characterized by strong hypertrophy of cell bodies and thickening of processes (group G2). These features are considered typical of reactive astrogliosis (Sofroniew & Vinters, 2010). The mechanisms involved in the brain damage induced by activated astrocytes are multiple, including oxidative stress by increasing the production of ROS (Thomas et al., 2004) and the release of proinflammatory cytokines (Gomez et al., 2007; Choi et al., 2015). In contrary, RP significantly reduced RT-induced reactive astrogliosis, yielding GFAP labeling similar to that of control animals with the independence of the RP formulation used.

TH is a rate-limiting enzyme involved in the synthesis of dopamine. Dopamine is synthesized in the dopaminergic neurons of the SNc area, stored in synaptic vesicles and released in response to stimuli in the striatum to exert its physiological function (Messripour & Mesripour, 2013). In PD patients, more than 70% of the dopaminergic neurons are dead leading to retraction of dopaminergic nerve terminals and depletion of dopamine in the striatum (Kudin et al., 2004; Duty & Jenner, 2011).

RT stimulation of ROS production is involved in dopamine redistribution from vesicles to the cytosol (Watabe & Nakaki, 2007) which blocks the transportation of dopamine from SNc to the striatum. Moreover, it has been indicated that decrease of antioxidant ability causes a clear loss of TH immunoreactivity in the striatum ipsilateral to the side of RT infusion (Saravanan et al., 2006). RT-induced PD in animals has been reported to be associated with an observable reduction of TH-immunoreactive neurons in the SNpc (Betarbet et al., 2000; Sherer et al., 2003; Bassani et al., 2014). In agreement with these studies, in our case, a marked reduction of TH immunoreactivity at the level of SNpc was observed (Figure 6(a)). In addition, when the quantitative analysis was carried out, the reduction of the fluorescence intensity at the level of the SNpc reached almost 30% (Figure 6(b)). More importantly, this decrease was completely reverted by RP-loaded PLGA NPs (group G3) but not benefit was obtained when the drug was given in saline (group G4).

## Conclusions

RP-loaded PLGA NPs have demonstrated to be effective in reverting neurodegeneration in an RT-induced animal model of PD.
